# Global Impact of COVID-19 on Nuclear Medicine Departments: An International Survey in April 2020

**DOI:** 10.2967/jnumed.120.249821

**Published:** 2020-09

**Authors:** Lutz S. Freudenberg, Diana Paez, Francesco Giammarile, Juliano Cerci, Moshe Modiselle, Thomas N.B. Pascual, Noura El-Haj, Pilar Orellana, Yaroslav Pynda, Ignasi Carrió, Stefano Fanti, Cristina Matushita, Ken Herrmann

**Affiliations:** 1 ZRN Rheinland and ZRN MVZ GmbH, Korschenbroich, Germany; 2 Department of Nuclear Medicine, University Hospital Essen, Essen, Germany; 3 Division of Human Health, International Atomic Energy Agency, Vienna, Austria; 4 PET/CT Department at Quanta Diagnostics and Therapy, Curitiba, Brazil; 5 KVNR Nuclear and Molecular Medicine, Pretoria, South Africa; 6 Philippine Nuclear Research Institute, Quezon City, Philippines; 7 Nuclear Medicine Department, Hospital Sant Pau, Barcelona, Spain; 8 Department of Experimental, Diagnostic and Specialty Medicine, S. Orsola Hospital University of Bologna, Bologna, Italy; and; 9 Instituto do Cérebro do Rio Grande do Sul, Porto Alegre, Brazil

**Keywords:** COVID-19, global impact, nuclear medicine, survey

## Abstract

The coronavirus disease 2019 (COVID-19) pandemic has placed significant challenges on health-care systems worldwide, whether in the preparation, response, or recovery phase of the pandemic. This has been primarily managed by dramatically reducing in- and outpatient services for other diseases and implementing infection prevention and control measures. The impact of the pandemic on nuclear medicine departments and their services has not yet been established. The aim of this online survey was to evaluate the impact of COVID-19 on nuclear medicine departments. **Methods:** A web-based questionnaire, made available from April 16 to May 3, 2020, was designed to determine the impact of the pandemic on in- and outpatient nuclear medicine departments, including the number of procedures, employee health, availability of radiotracers and other essential supplies, and availability of personal protective equipment. The survey also inquired about operational aspects and types of facilities as well as other challenges. **Results:** A total of 434 responses from 72 countries were registered and analyzed. Respondents reported an average decline of 54% in diagnostic procedures. PET/CT scans decreased by an average of 36%, whereas sentinel lymph-node procedures decreased by 45%, lung scans by 56%, bone scans by 60%, myocardial studies by 66%, and thyroid studies by 67%. Of all participating centers, 81% performed radionuclide therapies, and they reported a reduction of 45% on average in the last 4 wk, ranging from over 76% in Latin America and South East Asia to 16% in South Korea and Singapore. Survey results showed that 52% of participating sites limited their ^99m^Tc/^99^Mo generator purchases, and 12% of them temporarily cancelled orders. Insufficient supplies of essential materials (radioisotopes, generators, and kits) were reported, especially for ^99m^Tc/^99^Mo generators and ^131^I, particularly in Africa, Asia, and Latin America. **Conclusion:** Both diagnostic and therapeutic nuclear medicine procedures declined precipitously, with countries worldwide being affected by the pandemic to a similar degree. Countries that were in the postpeak phase of the pandemic when they responded to the survey, such as South Korea and Singapore, reported a less pronounced impact on nuclear medicine services; however, the overall results of the survey showed that nuclear medicine services worldwide had been significantly impacted. In relation to staff health, 15% of respondents experienced COVID-19 infections within their own departments.

Identified in December 2019, the severe acute respiratory syndrome coronavirus 2 (SARS-CoV-2) has since placed unprecedented challenges on countries worldwide to cope with the impact on health-care services (1,2). The preparedness of health-care systems varies greatly in countries and across regions as does the ability of these systems to accommodate large numbers of patients with severe coronavirus disease 2019 (COVID-19) (3–5).

Academic and hospital-based, private and public, inpatient and outpatient facilities, as well as diagnostic and therapeutic services, have been dramatically impacted by the pandemic (6); however, the fiscal and operational implications have not been elucidated and quantified yet.

A recent survey conducted in April 2020 in Austria, Germany, and Switzerland (7) suggested a mean reduction of PET/CT and conventional diagnostic nuclear medicine services ranging from 14% to 58%. Therapeutic services, especially for benign thyroid disorders and radiosynovectomies declined by 42% and 54%, respectively. In this regional study, the number of radioiodine therapies for thyroid cancer remained stable, suggesting that clinics continued to perform urgent interventions despite COVID-19 (7). One may assume that the impact of the pandemic would be comparable worldwide; however, countries’ preparedness and interventions to contain and mitigate the spread of COVID-19 as well as the availability of medical and financial resources vary between countries and regions. Reduced staff availability due to infection, and reluctance of patients to visit clinics out of concern of risking exposure to infection, may contribute to the observed COVID-19 impact on nuclear medicine services across the world. In addition, socioeconomic considerations and the resilience of health-care systems differ substantially from one country to another as well as within a country, which may result in a greater, or lesser, impact of COVID-19 observed in an area.

In cooperation with the International Atomic Energy Agency (IAEA), 2 authors of this article (Lutz S. Freudenberg and Ken Herrmann) conducted a worldwide survey with the aim to evaluate the impact of COVID-19 on nuclear medicine services across the globe and identify regional differences and challenges.

## MATERIALS AND METHODS

A web-based questionnaire was designed by 2 nuclear medicine specialists working in academia (Ken Herrmann) and private practice (Lutz S. Freudenberg). We attempted to evaluate the impact of the pandemic on inpatient and outpatient nuclear medicine operations, as well as on public versus private nuclear medicine departments. Survey questions addressed the following categories: operational aspects of nuclear medicine departments, impact on diagnostic and therapeutic nuclear medicine procedures, availability of personal protective equipment (PPE), and supply of radiotracers and other essential materials.

An interim analysis was performed on April 28, 2020. At that time, 223 participants had answered the survey, with many respondents indicating that the supply of radioisotopes and generators had been disrupted. To investigate this, an additional question was added to address supply challenges.

All questions were provided in English and placed on SurveyMonkey (https://www.surveymonkey.de/r/RHJMCKN) (Supplemental Appendix 1; supplemental materials are available at http://jnm.snmjournals.org). Invitations to participate in the survey were distributed by the IAEA and through personal networks. The survey was available from April 16 to May 3, 2020. All responses were checked for completeness and collected in an Excel (Microsoft) table. Some questions such as proportional reduction of radionuclide therapies were not applicable to all respondents and were therefore not answered by all. Responses to open-ended questions were collected separately.

Due to the heterogeneity of the data collected, we decided to perform only a descriptive analysis (see the “Limitations” section). Where applicable, we report mean and median results (as well as ranges where necessary). We present results along the lines of the above-mentioned main categories.

## RESULTS

### Participants

**FIGURE 1.**
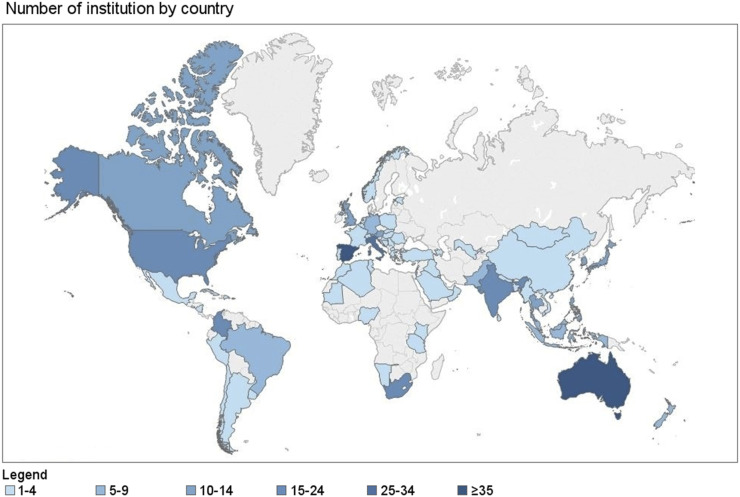
Participating nuclear medicine departments by country.

A total of 434 responses from 72 countries were recorded and made available for evaluation. Figure 1 shows the number of participants per country. Supplemental Figure 1 shows the continental distribution of participants.

On the basis of this analysis and geographic distribution as well as socioeconomic similarities we grouped the countries for subanalysis as follows: Italy, Spain (*n* = 88); Australia, New Zealand (*n* = 42); United States, Canada (*n* = 32); Thailand, Philippines, Indonesia (*n* = 32); Pakistan, India (*n* = 24); South Africa (*n* = 24); South Korea, Singapore (*n* = 16); and Colombia (*n* = 15). Eighty-five percent of the respondents were nuclear medicine specialists, 3% were radiologists, and 12% were others (mainly technologists and medical physicists). Forty-nine percent of the participants were university-based employees, 34% worked in community hospitals, and 17% were in private practice.

### Share of Outpatients

On average, 74.5% (median, 80%) of all services provided by participating centers are for outpatients. Sixty-eight percent of respondents reported a 52.6% decrease of outpatient visits in April 2020 (median, 50%). The center-based analysis shows an average decrease of 21% in the proportion of outpatients and a median decrease of 20%, during the COVID-19-crisis.

### Impact on Diagnostic Procedures

**FIGURE 2.**
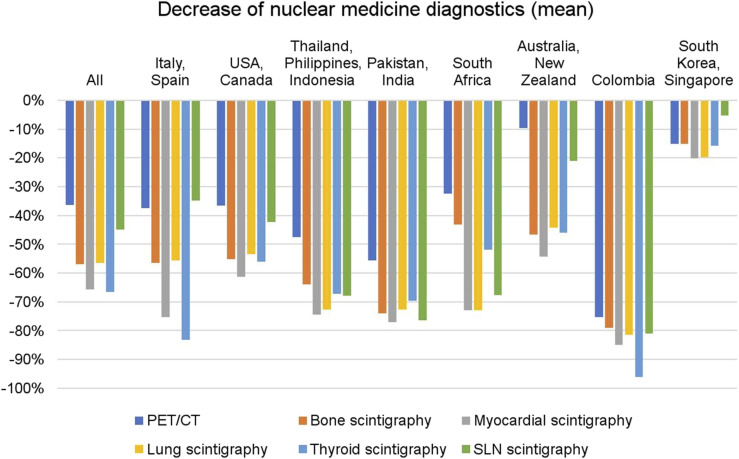
Decrease in diagnostics procedures globally and by regional subgroups.

Respondents reported an average decline of 54.4% in diagnostic procedures. PET/CT scans decreased by an average of 36%, whereas thyroid studies decreased by 67%, myocardial studies by 66%, bone scans by 60%, lung scans by 56%, and sentinel lymph-node procedures by 45%. Figure 2 shows the average decrease in diagnostic procedures globally and by regional subgroups

### Impact on Radionuclide Therapies

**FIGURE 3.**
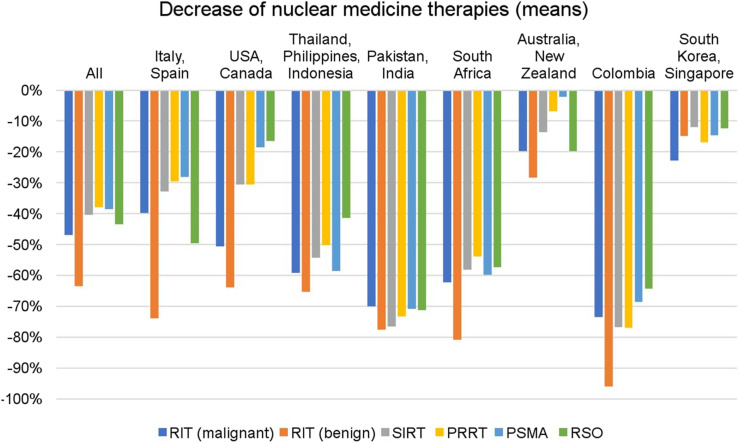
Decrease in nuclear medicine therapies globally and by regional subgroups.

Eighty-one percent of responding sites perform radionuclide treatments and observed a mean service reduction by 45% in April. Centers reported decreases in radioiodine therapy for thyroid cancer and benign diseases by an average of 47% and 63%, respectively, whereas radiosynovectomies decreased by 43%, selective internal radiation therapy by 40%, peptide receptor radionuclide therapy by 38%, and prostate-specific membrane antigen radioligand therapy by 38%, respectively. Figure 3 shows the decrease in therapeutic services stratified by geographic region.

### Employee Health and PPE

Fifteen percent of respondents experienced COVID-19 infections within their own departments: 12% reported that less than 20% of staff were infected, whereas 2.5% reported infection rates between 20 and 40%, and 0.5% observed high rates between 40 and 60%. Most infections occurred in Italy and Spain (28%), United States and Canada (16%), and Thailand, Indonesia, and Philippines (16%). No infections were reported in Colombia, India, Pakistan, Singapore, South Africa, and South Korea. Supplemental Figure 2 shows the percentage of COVID-19 infections in nuclear medicine staff.

As for the availability of PPE, 50% of the participants reported a shortage of PPE. Eighty-three percent of sites reported that stockpiles of PPE would last for only 1 mo, with no significant differences across geographic regions.

### Organizational Changes and Use of Communication Technologies

Fifteen percent of the respondents modified working hours for less than 20% of the staff (short, part-time. or staff turnover), 26% modified the work schedule between 20% and 70%, and 18% modified working hours by more than 70%. Staff transfer to other departments to meet special operational needs was reported in 34% of the sites.

Other operational adjustments as specified by 73% of respondents included online conferences (57%), online reporting (26%), and video consultations for patients and referring physicians (26%).

### Demand and Supplies of Materials

#### Demand

**FIGURE 4.**
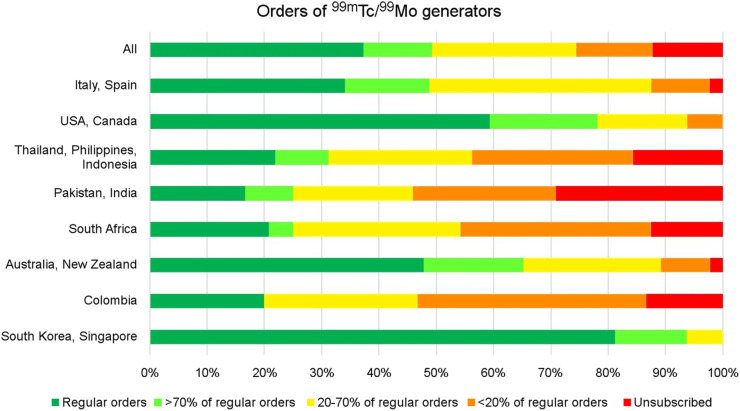
Impact in the orders of ^99m^Tc/^99^Mo during the COVID-19 pandemic.

Fifty percent of respondents reduced orders of ^99m^Tc/^99m^-molybdenum (^99m^Tc/^99^Mo) generators; of these, 12% maintained their orders for more than 70% of their regular demand, 25% maintained between 20% and 70% of their orders, and 13% maintained less than 20% of their orders. Another 12% canceled their generator orders entirely. The global impact and regional differences with respect to generator orders are shown in Figure 4.

#### Supply

**FIGURE 5.**
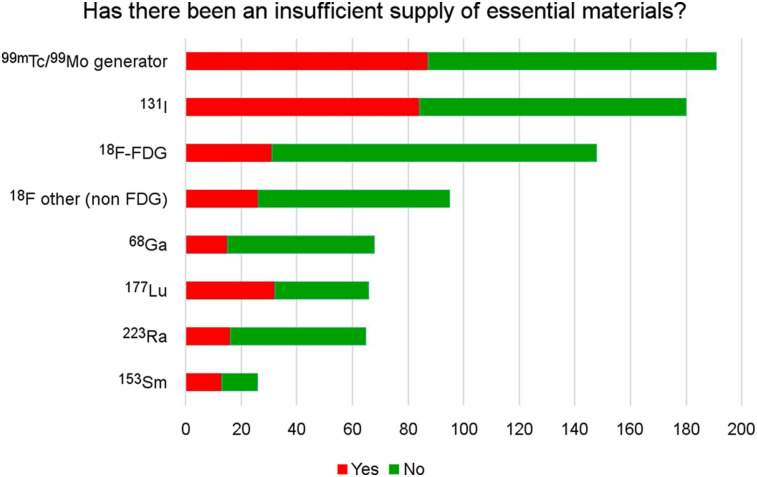
Disruption in the supply of essential materials. The *x*-axis denotates the number of participants.

Insufficient supplies of radioisotopes, generators, and kits were reported especially for ^131^I and ^99m^Tc/^99^Mo generators (Figure 5). The reduction of essential supplies varied substantially between regions and was more frequently reported from Africa, Asia, Oceania, and Latin America (Supplemental Figure 3).

## DISCUSSION

COVID-19 has affected health-care systems widely, and nuclear medicine departments are not the exception. Various factors contribute to the significant impact on the practice of nuclear medicine worldwide. While infection prevention and control (IPC) measures and postponement of nonemergent studies and other adaptative measures have been suggested in several publications (8–10), as well as a regional survey assessing the impact of COVID-19 on nuclear medicine services in 3 European countries that was conducted (7), at the time of our survey, there had been no global analysis of the impact of COVID-19 on nuclear medicine services. This lack of information encouraged us to conduct the survey and gain a better understanding of the challenges nuclear medicine departments are facing. A total of 434 responses from 72 countries confirmed significant reduction in nuclear medicine procedures: more than 50% in diagnostic and 40% in therapeutic procedures. This could be attributed to several factors such as changes in scheduling workflow with reduction in the number of appointments, reluctancy of patients to visit a medical center and be exposed to the risk of infection, deferral of nonurgent studies, deferral of surgeries and pre- or perioperative evaluations, shortages of essential supplies, implementation of IPC measures including social distancing and decrease in the numbers of health workers at one time to reduce staff exposure, and increase in the time assigned to each patient to include disinfection and cleaning procedures.

The decline in diagnostic tests was more pronounced in conventional nuclear medicine studies (thyroid, cardiac, bone, and lung scans) than for PET/CT scans. This may be for 2 reasons: first, PET tracers are produced by local cyclotrons, whereas most of the countries rely on international flights for the supply of ^99m^Tc/^99^Mo generators and other radioisotopes; and second, the more urgent nature for cancer assessments with PET/CT.

Among the respondents, it was found that countries and regions that were in the postpeak phase of the pandemic when they responded to the survey, such as South Korea and Singapore, reported less pronounced impact on diagnostic and therapeutic nuclear medicine procedures. However, on a global scale, it was found that all nuclear medicine services had been significantly and substantially impacted worldwide.

As for radionuclide therapies, the main reduction was reported in the radioiodine therapies for benign thyroid disease, with over 60%, followed by thyroid cancer (48%) and radiosynovectomies (43%), procedures that could be deferred for some weeks (8,9). Lesser declines were reported for selective internal radiation therapy, peptide receptor radionuclide therapy, and prostate-specific membrane antigen radioligand therapy.

Our data are in line with estimates from radiology practices (11–13), with expected decreases in study volumes “anywhere from 50–70%” (11). However, to date, no detailed radiologic surveys have been published. Other medical professions in the United States reported similar trends (14–18), with the downturn so severe that government funding programs have been initiated to provide financial support to medical facilities—including radiology (19). Without a doubt, the world economy faces serious challenges and although it is too early to assess the long-term impact that COVID-19 will have on health-care systems and on the practice of nuclear medicine, it is reasonable to assume that there will be differences between countries and regions (19,20).

Adoption of IPC measures are essential to protect health workers and patients while continuing to provide medical services (10). Thus, availability of PPE is critical. In our survey, 50% of participants reported shortage of PPE. Eighty-three percent of the sites reported that PPE stockpiles would last for only 1 mo, with no significant differences among geographic regions.

COVID-19 infections in staff were reported in 15% of responding centers. The highest rates were reported in Spain and Italy, countries that also had the highest number of COVID-19 cases at the time of the survey.

Nuclear medicine relies on complex supply chains and advanced logistics. The lockdowns imposed by most countries and the closure of borders, including flights (21), have generated shortages of radionuclides and other essential supplies in many countries. Insufficient supplies of ^99m^Tc/^99^Mo generators affected mainly Latin America (70%), Asia (60%), and Africa 48%).

Availability of ^131^I for radioiodine therapy was also significantly impacted in Latin America (60%), Asia (55%), Africa (52%), and Oceania (50%), contributing to a steep decline in therapies in these regions. According to the IAEA, producers of medical radioisotopes continue to operate with some adjustments, and medical radioisotopes and radiopharmaceuticals have been recognized as “essential services” in many countries. However, there are significant disruptions of the supply chains due to the limitation in transportation (22).

The overall significant decrease in nuclear medicine procedures also resulted in a reduction of working hours in 59% of the surveyed centers, affecting large numbers of staff. This reduction may lead to significant socioeconomic impact in several countries (18). We are now looking at varying degrees of preparedness for countries to ramp up operations as some regions are currently recovering from the pandemic, other regions have plateaued, and some regions still face increasing numbers of infections. It will take time to assess whether we are prepared to restart operations safely (23).

## LIMITATIONS

Although we obtained responses from 434 centers in 72 countries, which could be considered a small sample of the existing nuclear medicine centers worldwide, our data provide a global perspective of the impact of COVID-19 on nuclear medicine services. We obtained 100% situational representation of nuclear medicine services from some countries with few nuclear medicine centers, such as Mauritius (1) and Mauritania (2). On the other hand, for most participating countries, there was limited representation when comparing the number of responding centers with those registered in IMAGINE, the IAEA’s medical imaging and nuclear medicine global resources database (24). For example, only 2 of 45 centers from Chile, 15 of 93 centers from Colombia, and 15 of 300 nuclear medicine departments in India participated in the survey. This is the reason why only a descriptive analysis of the collected data was performed. Weighted distribution of respondents by continents according to the availability of SPECT per 1 million inhabitants registered in the IMAGINE database of the IAEA (24) is represented in Supplemental Figure 4.The current survey cannot differentiate whether reduced numbers of nuclear medicine studies and interventions are due to the patient’s preference to postpone or cancel studies due to safety concerns; the department’s preference to reduce study numbers due to safety concerns; the limited supplies of kits, radioisotopes, and generators; or some or all of the above.

Another important limitation is that the survey did not address the problems, challenges, and consequences for medical training (25), residency (26–28), and research (29,30).

Moreover, this survey has a limitation in that there is an overrepresentation of certain countries and regions. This survey aimed to provide a global situational snapshot of the COVID-19 impact on nuclear medicine services. A follow-up survey to better assess the long-term impact of CODIV-19 in nuclear medicine centers is required. It is important to monitor the restitution of the supply chains of radioisotopes, generators, and other essential materials, as well as the socioeconomic impact on nuclear medicine departments.

## CONCLUSION

Both diagnostic and therapeutic nuclear medicine procedures declined precipitously with the pandemic, affecting countries around the world to a similar degree. Countries that were in the postpeak phase of the pandemic when they responded to the survey were found to report a less pronounced impact on nuclear medicine services.

It is unknown whether the decrease in the implemented procedures is attributable to patients’ fears and preferences, safety precautions adopted by nuclear medicine centers, disruption of supply chains and logistical challenges, or a combination of all of the above.

It is our responsibility to continue providing essential services to ascertain adequate diagnostic and therapeutic patient services, while ensuring proper IPC measures, thus safeguarding the health of staff, patients, and the public. It is also important to address the significant disruptive economic impact of this pandemic on health-care systems in general and on nuclear medicine services in particular. The more we know about the current and upcoming challenges, the better we can learn and adapt collectively to them.

## DISCLOSURE

No potential conflict of interest relevant to this article was reported.
