# Supplementary material for: Global Impact of COVID-19 on Nuclear Medicine Departments: An International Survey in April 2020
Source: J Nucl Med. 2020 Sep;61(9):1278–83. doi: 10.2967/jnumed.120.249821 (PMC7456173; doi:10.2967/jnumed.120.249821)
Supplement: Supplementary file 1 [file jnm249821SupplementaryData.pdf]

Supplemental figures

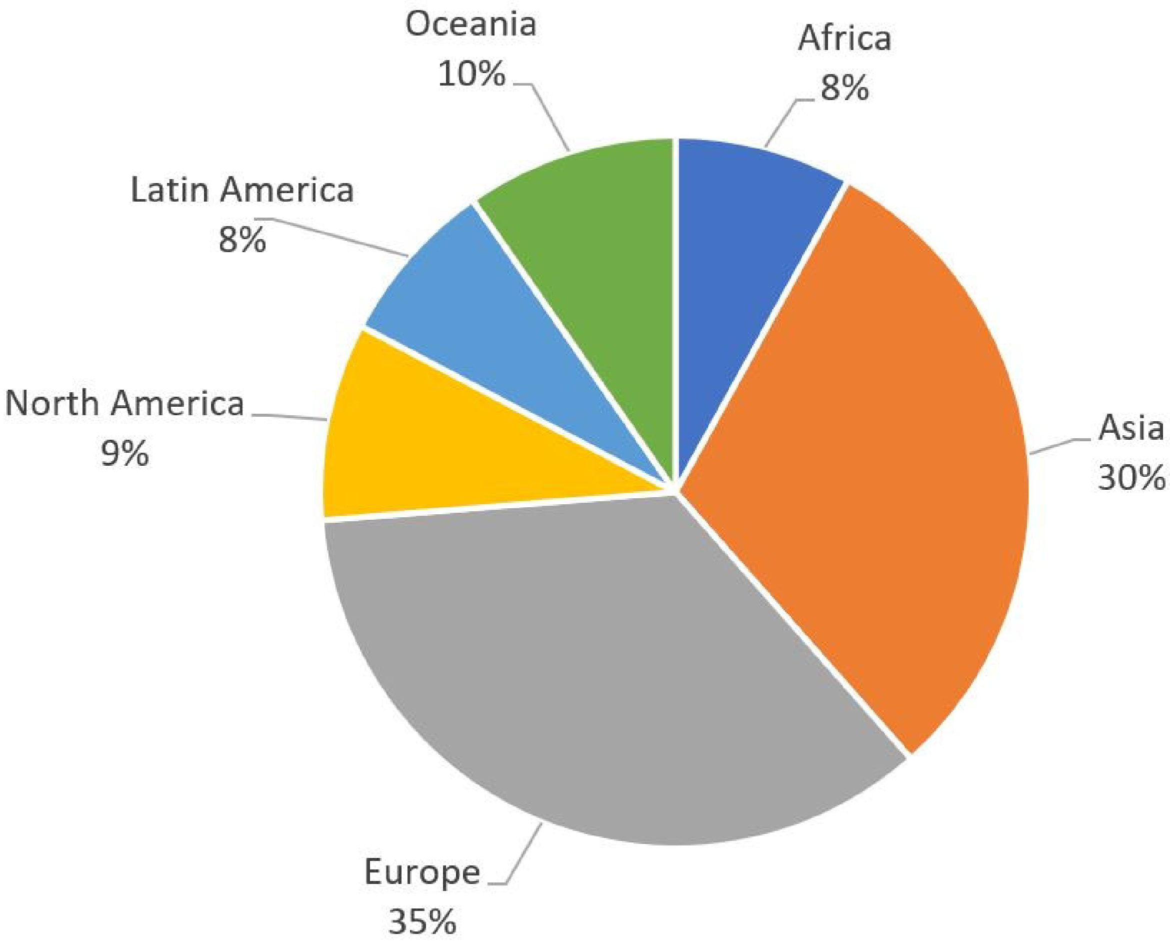

Supplemental Figure 1: Distribution of respondents on the continents (n=434)

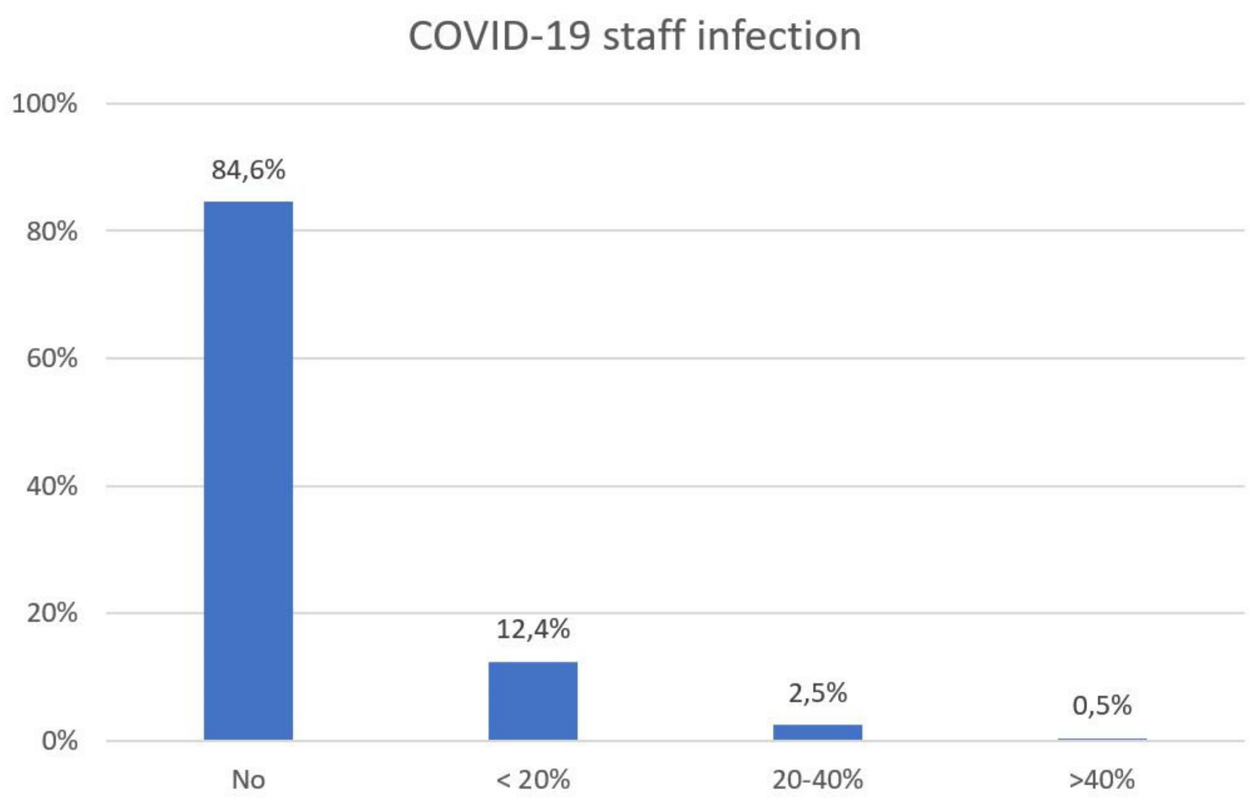

Supplemental Figure 2: Percentage of COVID-19 infections in nuclear medicine staff

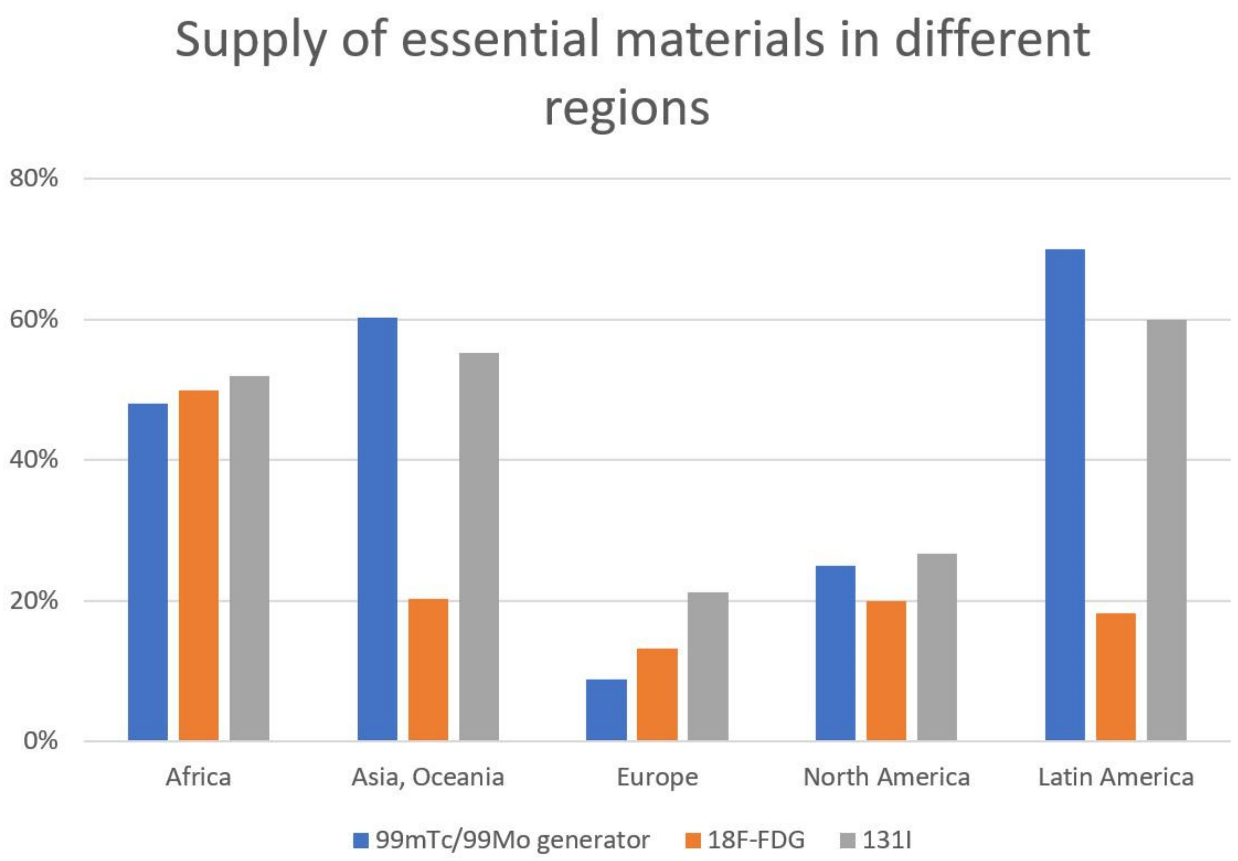

Supplemental Figure 3: Reduction of essential supplies in different regions

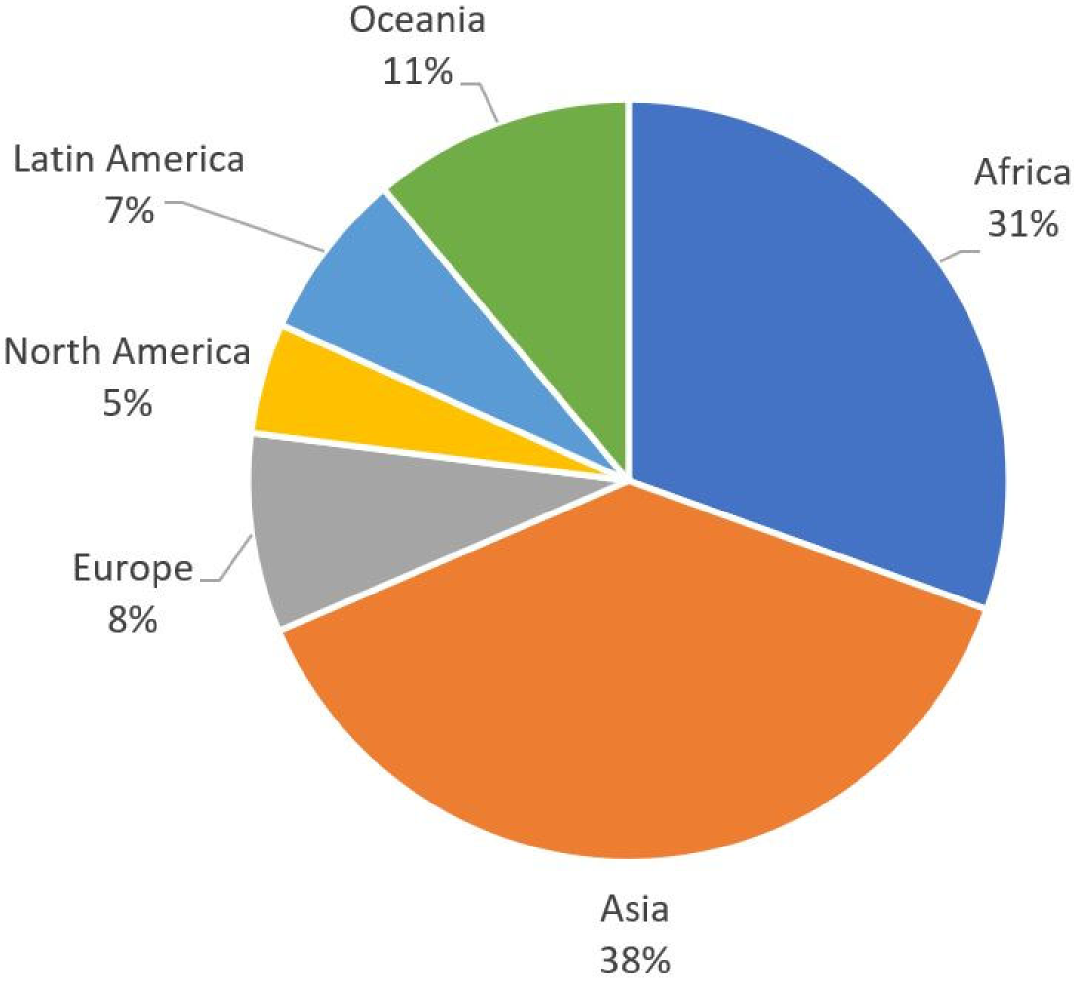

Supplemental Figure 4: Weighted distribution of respondents by continents according to the availability of SPECT / 1 million inhabitants registered in the IMAGINE database of the IAEA [24]

Appendix

Survey “Influence of COVID-19 on Nuclear Medicine Departments International Survey”

Influence of COVID-19 on Nuclear Medicine Departments International Survey

Dear Colleague,

The current COVID-19 crisis also has an impact on the care of nuclear medicine patients and the operation of nuclear medicine practices and clinics. These effects appear to be different due to the geographical location and structural conditions.

Together with the IAEA we would like to record the current effects, evaluate them nd make them available to the members. The higher the participation rate, the more meaningful we get to third parties, such administrations, health officials, etc. Therefore, we would like to ask you to take 5 minutes to answer this online questionnaire.

We thank you for your support.

With best regards!

Lutz Freudenberg and Ken Herrmann

**Organization**	
1. Country	…………………………………………………………………………………
2. City	…………………………………………………………………………………
3. Where do you practice?	☐ University Hospital<br>☐ Hospital<br>☐ Private practice
4. Your expertise	☐ Nuclear medicine specialist<br>☐ Radiologist<br>Other ……………………………………………………………………………
5. Function or role in your department	☐ Head of department<br>☐ Consultant<br>☐ Resident<br>Other ……………………………………………………………………………
**Nuclear Medicine DIAGNOSTIC**	
6. What is the “normal” proportion of outpatients in your department?	☐ 0% - no outpatients in my department.<br>☐ 10% ☐ 20% ☐ 30% ☐ 40%<br>☐ 50% ☐ 60% ☐ 70% ☐ 80% ☐ 90%<br>☐ 100% - only outpatients in my department
7. During the COVID-19 crisis: Was there a shift in the relationship between outpatients and stationary patients?	☐ Yes<br>☐ No
8. If yes: What is the current proportion of outpatients and stationary patients?	☐ 0% - no outpatients in my department.<br>☐ 10% ☐ 20% ☐ 30% ☐ 40%<br>☐ 50% ☐ 60% ☐ 70% ☐ 80% ☐ 90%<br>☐ 100% - only outpatients in my department
During the COVID- 19 crisis: Did the number of nuclear medicine **diagnostic** procedures change? Please specify	
9. PET/CT	-100% ├──────●──────┤ 100%
10. Bone scintigraphy	-100% ├──────●──────┤ 100%

11. Myocardial scintigraphy	-100% 100%
12. Lung scintigraphy	-100% 100%
13. Thyroid scintigraphy	-100% 100%
14. Sentinel lymph-node scintigraphy	-100% 100%
15. Others	…………………………………………………………………………………………
**Nuclear Medicine THERAPY**	
16. Do you regularly perform nuclear medicine therapies in your department?	☐ Yes<br>☐ No
**If YES**: During the COVID-19 crisis, did the number of nuclear medicine therapies change? **Please specify.**	
17. Thyroid (malignant)	-100% 100%
18. Thyroid (benign)	-100% 100%
19. Selective internal radiation therapy (SIRT)	-100% 100%
20. Peptide receptor radionuclide therapy (PRRT)	-100% 100%
21. Prostate-specific membrane antigen (PSMA)	-100% 100%
22. Radiosynoviorthesis (RSO)	-100% 100%
**Personal Protective Equipment (PPE)**	
23. Is there a shortage of personal protective equipment in your department for you and your employees?	☐ Yes<br>☐ No
24. For how many days do you have personal protective equipment on stock for you and your employees?	☐ Less than 2 days<br>☐ 2-7 days<br>☐ 7-14 days<br>☐ 14-28 days<br>☐ more than 28 days
**Employee Health Organizational Adjustments to the COVID-19 Crisis**	
25. Have employees in your department been infected with the new Corona virus?	☐ No<br>☐ Yes, less than 20% of the employees<br>☐ Yes, 20% - 40% of the employees<br>☐ Yes, 40% - 60% of the employees<br>☐ Yes, more than 60% of the employees
26. Have employees of your department been transferred to other clinical departments and areas?	☐ No<br>☐ Yes, less than 20% of the employees<br>☐ Yes, 20%- 40% of the employees<br>☐ Yes, 40%- 60% of the employees<br>☐ Yes, more than 60% of the employees
27. Have employees to work in short time?	☐ No<br>☐ Yes, less than 20% of the employees<br>☐ Yes, 20%-70% of the employees<br>☐ Yes, more than 70% of the employees
28. Do you use online tools in your department you have not used before?	☐ No<br>☐ Yes, online conferences<br>☐ Yes, online reporting<br>☐ Yes, video consultations for patients and referring physicians<br>☐ Others ………………………………………………………………………

29. Have you taken other measures to deal with the crisis?	…………………………………………………………………………………………
**Supply of Mo/ Tc an other essential materials**	
30. Did you adjust your orders for Mo/Tc generators?	☐ No<br>☐ Yes, we are still ordering >70% of our normally ordered activity<br>☐ Yes, we are still ordering 20-70% of our normally ordered activity<br>☐ Yes, we are still ordering less than 20% of our normally ordered activity<br>☐ Yes, we have unsubscribe our generators.
31. At any time during the pandemic, has the institution's <u>supply</u> of the following essential materials been <u>insufficient</u>?	
31.1. Mo/Tc generators	☐ Yes<br>☐ No<br>☐ Not applicable
31.2. 18 F-FDG	☐ Yes<br>☐ No<br>☐ Not applicable
31.3. Other 18 F labelled tracers	☐ Yes<br>☐ No<br>☐ Not applicable
31.4. Gallium 68 Generators	☐ Yes<br>☐ No<br>☐ Not applicable
31.5. Iodine 131	☐ Yes<br>☐ No<br>☐ Not applicable
31.6. Lutetium 177	☐ Yes<br>☐ No<br>☐ Not applicable
31.7. Radium 223	☐ Yes<br>☐ No<br>☐ Not applicable
31.8. Samarium 153	☐ Yes<br>☐ No<br>☐ Not applicable
31.9. Cold kits	☐ Yes<br>☐ No<br>☐ Not applicable
31.10. Others	…………………………………………………………………………………………
32. Do you have other/ other ideas for dealing with the effects of the COVID-19 crisis on nuclear medicine that you want to share with us?	…………………………………………………………………………………………
33. Do you want to keep in touch with us? If yes, please share your email.	…………………………………………………………………………………………
